# Temperature-Driven Structural Evolution during Preparation of MCM−41 Mesoporous Silica

**DOI:** 10.3390/ma17081711

**Published:** 2024-04-09

**Authors:** Tao Xu, Kuixin Cui, Shengming Jin

**Affiliations:** 1 School of Minerals Processing and Bioengineering, Central South University, Changsha 410083, China; xu-tao@mail.csu.edu.cn (T.X.); kuixin.cui@csu.edu.cn (K.C.); 2 Key Laboratory for Mineral Materials and Application of Hunan Province, Central South University, Changsha 410083, China

**Keywords:** silica, mesoporous aperture, structural order, micelle aggregation state, thermal expansion and contraction

## Abstract

This study explores the influence of micelles on the evolution of MCM−41’s pore structure via 24 h hydrothermal treatments in a range of temperatures from 100 °C to 200 °C. MCM−41 was characterized using BET, SAXD, FTIR, TEM, and TG-DSC. The findings demonstrate that with temperature elevation from 100 °C to 160 °C, the micelles undergo expansion, leading to an enhanced lattice constant from 4.50 nm to 4.96 nm and an increase in pore diameter from 3.17 nm to 3.45 nm, while maintaining the structural orderliness of the pore channels. Upon cooling, the reversible contraction of micelles and the strategic addition of water glass contribute to a reduction in pore size. However, at a threshold of 180 °C, the SAXD (100) peak’s half-peak width surges by approximately 40% relative to that at 160 °C, illustrating a progressive disruption of the hexagonal configuration of MCM−41. Coupled with elevated silica dissolution at higher temperatures in an alkaline solution, a total disintegration of the ordered pore structure at 200 °C results in a drastic reduction in the specific surface area to 307 m^2^/g. These results are beneficial to developing structural transformation mechanisms of MCM−41 materials and designing mesoporous materials via temperature modulation innovatively.

## 1. Introduction

Since the seminal discovery of MCM−41 (the 41st in Mobil Composition of Matter) type mesoporous silica by Kresge and colleagues [1] from Mobil Oil Corporation in 1992, this material has captivated the materials science and chemical engineering communities due to its distinctive pore architecture, extensive surface area, adjustable pore diameters, and rich surface hydroxyl groups. Researchers have employed synthesis methods such as sol-gel [2,3], hydrothermal [4], and microwave-assisted [5] techniques to extensively explore various factors affecting the pore size, wall stability, and surface properties of MCM−41 materials [6,7,8,9]. By adjusting synthesis conditions, researchers have successfully tailored the mesoporous structure and chemical properties of MCM−41, unlocking its vast potential in diverse fields like adsorption [10,11], catalysis [12,13,14,15], drug delivery [16], and environmental remediation [17].

The synthesis mechanisms of mesoporous materials predominantly encompass the cooperative self-assembly and liquid crystal templating theories. The cooperative self-assembly mechanism involves interactions between surfactant micelles and oligomeric silicates through electrostatic forces, hydrophobic interactions, and van der Waals forces [18], culminating in orderly structures. Monnier et al. [19] proposed a mechanism transitioning from lamellar to hexagonal phases, effectively elucidating the hexagonal pore formation in MCM−41. Beck [20] introduced the liquid crystal templating mechanism, grounded in prior research on surfactant aggregation in various solvents, where surfactants were observed to spontaneously form a two-dimensional hexagonal liquid crystal phase at certain concentrations. This mechanism posits that oligomeric silicates condense within an existing liquid crystal phase, thereby constructing the mesoporous material’s framework. Several researchers have simulated the synthesis process of MCM−41 using simulation software like Gromacs, with results lending further support to the cooperative self-assembly mechanism [21,22]. However, these findings still require empirical validation through experimental studies.

Recent advancements in material synthesis techniques, coupled with a deeper understanding of reaction mechanisms, have enabled the manipulation of MCM−41’s pore size and wall thickness by modulating synthesis conditions. This adaptability meets the diverse requirements of various fields. For instance, petroleum cracking catalysis at temperatures above 500 °C necessitates thicker pore walls to ensure higher thermal stability. Conversely, in drug delivery applications, the pore size is tailored to the size of drug molecules, with a reduced wall thickness to increase porosity and thus enhance drug loading rates [23,24]. Therefore, studying the synthesis conditions that affect the structure of the final product is crucial for tailoring MCM−41 materials to specific applications. Researchers have demonstrated that modifying the surfactants’ hydrophobic chain length and incorporating specific organic additives like 1,3,5-trimethylbenzene (TMB) and N,N-dimethylhexadecylamine (DMHA) have facilitated pore size adjustments within the 2–10 nm range [20,25]. Khushalani et al. [26] discovered that hydrothermal timing could regulate pore size, observing an increase in pore diameter from 2.8 nm to 3.4 nm under 150 °C hydrothermal treatment. Cheng and associates [27] further observed that with elevated temperatures and extended durations, MCM−41’s pore size could be augmented from 2.6 nm to 3.7 nm, and wall thickness from 1.3 nm to 2.7 nm, noting enhanced thermal stability with thicker walls. Nonetheless, the mechanisms underlying the enlargement of mesopores and the structural collapse at elevated hydrothermal temperatures remain somewhat elusive, necessitating distinct discussions for varying synthesis methodologies.

This study predominantly concentrates on the impact of hydrothermal temperature on the mesostructure of MCM−41 synthesized using water glass as the silicon source, exploring the inherent link between surfactant micelle aggregation patterns and the material’s structure. Experimental observations reveal that as the hydrothermal crystallization temperature increases from 100 °C to 160 °C, there is a noticeable enlargement in the pore size of MCM−41, accompanied by an expansion of its lattice structure while maintaining a high degree of structural order. However, a further temperature increase to 180−200 °C precipitated a marked decline in pore channel orderliness, culminating in the vanishing of mesopores. By integrating insights from micelle aggregation studies and considering the dynamics of the silica precipitation–dissolution equilibrium, this research probes into the complex reaction mechanisms that govern these structural transformations.

## 2. Materials and Methods

### 2.1. Materials

The water glass used in this study, obtained from Fujian Sanming Zhengyuan Chemical Co., Ltd., in Sanming, China, had a modulus of 3.45, a concentration of 336 g/L, and contained impurities of 0.019% Fe and 0.097% Al. The cationic surfactant, cetyltrimethylammonium bromide (CTAB), was sourced from Adamas-Beta Reagents, Shanghai, China, with a purity of ≥99%. Concentrated sulfuric acid, obtained from Hunan Changsha Huihong Reagent Co., Ltd., in Changsha, China, had a purity of ≥98%. The ultrapure water was self-produced using laboratory equipment, achieving a resistivity of 18.25 MΩ/cm. All materials were used directly without further processing.

### 2.2. Preparation and Characterization Methods

Initially, 64 mL of deionized water was heated to 60 °C in a beaker. Subsequently, 5.76 g of CTAB powder was gradually added under stirring at 400 rpm, allowing 30 min for complete dissolution. Subsequently, we adjusted the stirring speed of the solution to 520 rpm and slowly added 14 mL of the water glass solution using a peristaltic pump at a rate of 2.5 mL/min. After addition, the suspension was sealed to prevent evaporation and stirred for 3 h at 60 °C. The pH was then adjusted to 9, followed by an additional 10 min of stirring. The molar ratio of the raw materials was SiO_2_:CTAB:HO = 1:0.2:54. The suspension was transferred to a cleaned and dried 120 mL polytetrafluoroethylene (PTFE) liner, placed inside a stainless-steel hydrothermal autoclave. After undergoing hydrothermal treatment for 24 h at the predetermined temperature, the sample was thoroughly washed with ample water until it reached a neutral pH. Subsequently dried at 80 °C for a minimum of 8 h, the product underwent calcination at a rate of 2 °C/min up to 550 °C for 6 h to eliminate CTAB. The calcined samples are distinguished by the hydrothermal synthesis temperature T, and the uncalcined samples are represented by u−T.

Small-angle X-ray diffraction (SAXD) analyses were performed on Dandong Tongda TD3500 X-ray diffractometer equipped with Cu-Kα radiation (35 kV, 30 mA), 0.01° step size, and 0.5 s scanning speed. The specific surface area and pore size distribution were measured at liquid nitrogen temperature using nitrogen as the adsorbate on the Jingwei Gaobo JW−ZQ200. Prior to analysis, samples were degassed under a vacuum at 200 °C for over 2 h. The Brunauer–Emmett–Teller (BET) equation was applied for the specific surface area calculation within a relative pressure range of 0.05−0.25, and the mesopore size distribution was assessed using the Barrett–Joyner–Halenda method with Kruk–Jaroniec–Sayari correction for cylindrical mesopores (BJH-KJS) method from the absorption branch. The total pore volume was calculated based on the amount of nitrogen adsorbed at a P/P_0_ of 0.99. Fourier-transform infrared spectroscopy (FTIR) measurements were performed on a Thermo Fisher Scientific Nicolet iS50 FTIR spectrometer, with samples dried at 80 °C for more than 6 h prior to testing. Thermogravimetry-differential scanning calorimetry (TG-DSC) was conducted on a NETZSCH STA 449 F3 Jupiter thermal analyzer. The analyses were conducted over a temperature range of 30 to 800 °C, with a heating rate of 5 °C/min, under an atmosphere composed of 50 mL/min of air and 20 mL/min of argon. Finally, the pore channel structure of the samples was observed using a Thermo Fisher Scientific Talos F200i transmission electron microscope (TEM) at 200 kV.

## 3. Results and Discussion

**Figure 1:**
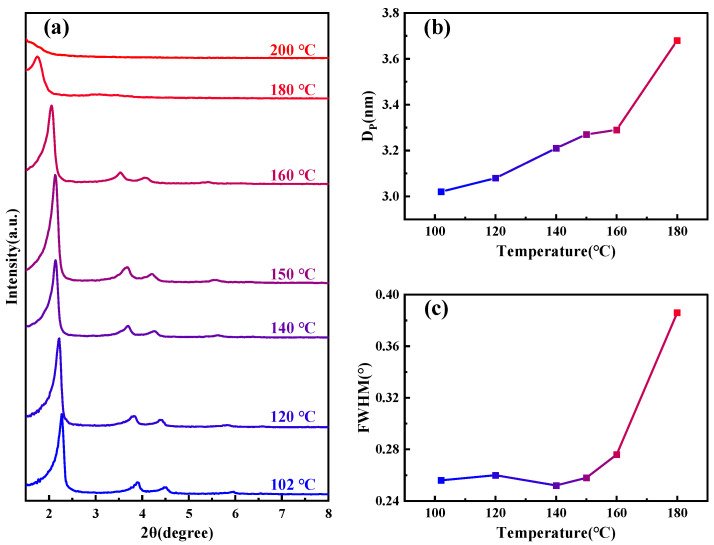
(**a**) SAXD spectra of samples under various temperature conditions; (**b**) variation of sample pore size under different temperature conditions; and (**c**) changes in the half-width of the (100) peak for samples at different temperature conditions.

Figure 1a shows SAXD patterns of the samples subjected to different hydrothermal crystallization temperatures, the details of which are outlined in Table 1. As the temperature increases, the (100) crystal plane spacing of MCM−41 continuously expands, signifying ongoing lattice expansion. Notably, above 180 °C, the diffraction peaks of the material disappear rapidly. From the change in pore size of the material shown in Figure 1b, as the temperature increases, the pore size also gradually becomes larger. The half-width variations of the small-angle X-ray (100) diffraction peaks at different temperatures, as depicted in Figure 1c, allow us to approximate the specific changes in structural orderliness, a higher half-width indicates poorer orderliness. Between 100–160 °C, the orderliness remains relatively stable, but it sharply declines upon reaching 180 °C, and the material loses order completely when the temperature continues to rise to 200 °C. Similar diffraction peak disappearance phenomena have been observed by other researchers in studies related to MCM−41’s properties [28,29].

**Figure 2:**
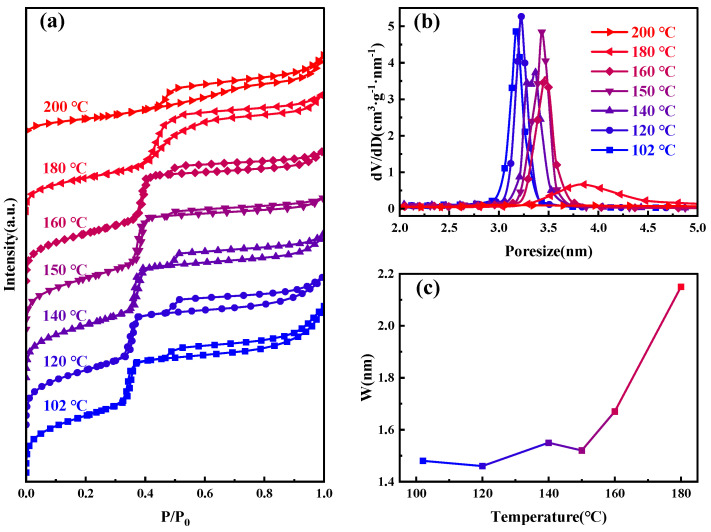
Samples at different hydrothermal crystallization temperatures (**a**) nitrogen adsorption isotherms; (**b**) the mesopore size distribution curves; and (**c**) variations in pore wall thickness.

Figure 2 presents the nitrogen adsorption–desorption isotherms, and BJH−KJS pore size distribution graphs of MCM−41 materials synthesized and calcined at different temperatures, with specific surface area and pore size data detailed in Table 1. According to the gas adsorption isotherm classification by the International Union of Pure and Applied Chemistry (IUPAC), the observed curve type is Type IV [30]. When the hydrothermal temperature is between 100–160 °C, the curves of the samples are very similar. The H1 hysteresis loop observed between P/P_0_ of 0.3 and 0.4 suggests capillary condensation, indicative of a material with centrally distributed mesopores. The figure shows the step region progressively moving rightward, signifying the continuous enlargement of the material’s mesopore pore size. It can also be seen from the figure that an additional H4-type hysteresis loop appears at P/P_0_ between 0.45–1, indicating the presence of larger pores within the material [31,32]. During desorption, the smaller mesopores hinder the evaporation of liquid nitrogen inside these large pores, making the desorption pressure much smaller than the adsorption pressure [33]. The nitrogen adsorption amount of the material at P/P_0_ between 0.8–1.0 increases sharply, indicative of the typical stacking pores formed by the accumulation of silica particles. Moreover, as the increase in hydrothermal crystallization temperature, this adsorption enhancement becomes less pronounced. This suggests that the silica particles gradually merge under high-temperature hydrothermal conditions, and adsorption from stacking pores gradually decreases [34,35]. After 180 °C hydrothermal treatment, the sample’s pore size continues to grow, the H1 type hysteresis loop shifts rightward, and the presence of larger pores causes this loop to merge with the right-side H4-type hysteresis loop, ultimately forming this atypical hysteresis loop. After 200 °C hydrothermal treatments, the H1 type hysteresis loop disappears, signifying the destruction of the material’s mesoporous channels, with the material’s stacking pores and inherent voids constituting the H4 type hysteresis loop in its adsorption isotherm.

Concerning the gradual increase in pore size and loss of order with increasing hydrothermal temperature, we performed FTIR spectroscopy tests on the uncalcined MCM−41 material to rule out CTAB decomposition. The results, as shown in Figure S1a, indicate almost identical infrared spectroscopy peaks across the materials, suggesting that the CTAB did not decompose. The pH of the MCM−41 material synthesized in this experiment was only 9.0, and the pH of the solution after hydrothermal heating was approximately 10.3. To verify whether CTAB decomposes after hydrothermal heating at high temperatures in the experimental environment, we designed the following experiment: NaOH solution was added to the CTAB solution prepared for synthesizing MCM−41, adjusting the pH to 11, followed by hydrothermal treatment at 180 °C and 200 °C for 24 h. After drying the resultant solution at 80 °C, they were analyzed using FTIR Spectroscopy. As shown in Figure S1b, both the hydrothermally treated and untreated CTAB demonstrated substantial similarity, indicating that there was no decomposition of CTAB under the experimental conditions. This finding aligns with the experimental results of Khushalani et al. [26], who used ^1^H Nuclear Magnetic Resonance (NMR) to analyze the organic compounds extracted from MCM−41 and observed that CTAB did not decompose at 150 °C.

It is well-known that an increase in temperature intensifies molecular thermal motion. During the temperature rise, surfactant molecules in micelles tend to escape into the aqueous solution, increasing the entropy of the overall solution system. According to the Tanford formula [36]:

The volume of the surfactant head group

(1) $$V_{0}=\left[27.4+26.9\left(n_{c}-1\right)\right]{2Å}^{3}$$

and the maximum chain length

(2) $$l_{c}=[1.54+1.26\left(n_{c}-1\right)]Å,$$

where n is the number of carbon atoms in the carbon chain, we calculated the head group volume V_0_ of CTAB to be 861.8 Å^3^, and the chain length lc to be 20.44 Å. If CTAB molecules were fully extended and arranged into cylindrical rod-like micelles, their diameter should be 2$l_{c}$ ≈ 40.88 Å. However, many researchers have synthesized MCM−41 pore sizes smaller than 4 nm without any additives [31,37,38]. These results suggest that the surfactant molecules in the micelles are not fully extended. Based on surface tension and conductivity tests of CTAB solutions by Shah et al. [39,40], and our extended experiments at higher temperatures, as shown in Figure S2a, we obtained the variation of the critical micelle concentration (CMC) with temperature, as illustrated in Figure S2b. It is evident that the CMC concentration gradually increases, with more surfactant molecules dissolving in water, thereby reducing the number of molecules forming the micelles. If CTAB maintains its length within the micelle while the micelle diameter increases, it contradicts the experimental results, as an increase in internal molecular number is required [41]. Therefore, the reason for the gradual increase in MCM−41 pore size with temperature is the thermal expansion of the micelles. During the hydrothermal crystallization process, the following precipitation–dissolution equilibrium exists [42,43]:

(3) $$\begin{array}{c} \equiv Si\text{-}O\text{-}Si\equiv +{\;H}_{2}O\overset{\mathrm{Heat}}{\Leftrightarrow }2\;\equiv \mathrm{Si}\text{-}\mathrm{OH} \end{array}$$

Heating promotes the condensation of oligomeric silicates, raising the solution pH and causing the expanded micelles to affect the precipitation–dissolution equilibrium at the interface. Dissolved oligomeric silicates re-condense on the micelle surface, ultimately forming silica channels matching the micelle diameter. Additionally, the shielding effect of $\equiv \mathrm{Si}\text{-}O^{-}$ at the interface on the micelle surface charge results in a small difference in micelle spacing, reflected in the relatively constant pore wall thickness, as shown in Figure 2c.

**Figure 3:**
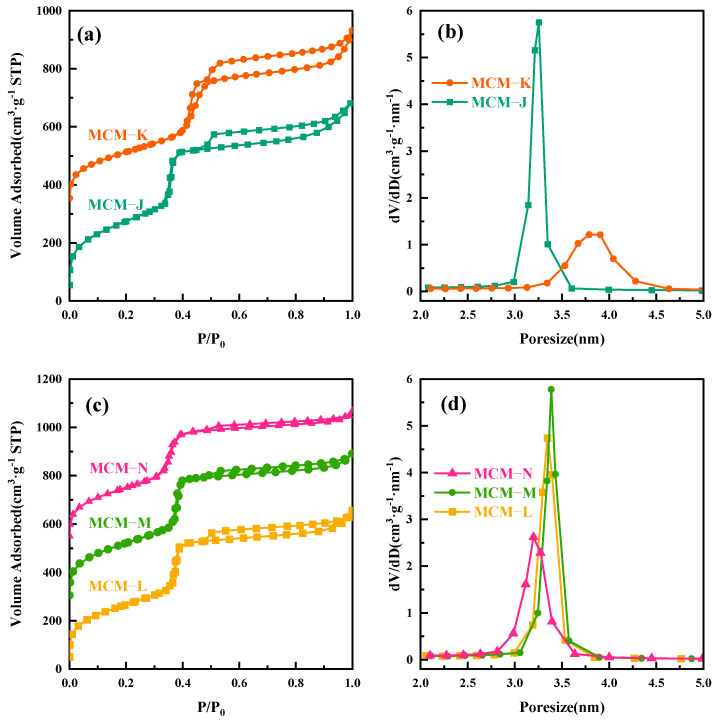
(**a**) Nitrogen adsorption isotherms of MCM−J and MCM−K; (**b**) the mesopore pore size distribution curves for MCM−J and MCM−K; (**c**) nitrogen adsorption isotherms of MCM−L, MCM−M, and MCM−N; and (**d**) the mesopore pore size distribution curves for MCM−L, MCM−M, and MCM−N.

To further elucidate the thermal expansion and contraction of micelles with temperature changes, we conducted the following experiment. The MCM−41 material, hydrothermally treated at 120 °C for 24 h and labeled as MCM−J, was directly subjected to a secondary hydrothermal crystallization treatment at 180 °C for another 24 h, resulting in a sample labeled MCM−K. All the labeled samples are given in Table 2. The surface area and mesopore analysis results are shown in Figure 3a,b. As the hydrothermal temperature increased to 180 °C, the mesopore pore size expanded from 3.25 nm to 3.78 nm. However, since MCM−K had already crystallized for 24 h at 120 °C, the high degree of silica condensation slowed down the precipitation–dissolution rate and hindered the pore enlargement speed, ultimately resulting in a pore size smaller than that obtained by direct hydrothermal treatment at 180 °C for 24 h. As depicted in Figure S3a, the SAXD analysis reveals the almost complete attenuation of the diffraction peaks corresponding to the (110) and (200) crystallographic planes, coupled with a marked expansion in the interplanar spacing of the (100) plane, aligning well with the inferences drawn in our study.

**Figure 4:**
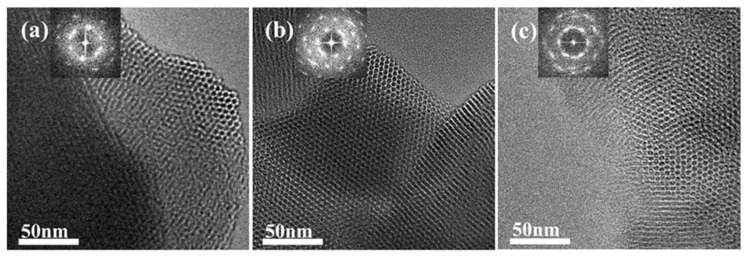
TEM image of the samples (**a**) MCM−L; (**b**) MCM−M; and (**c**) MCM−N. The top-left corner of each image shows the corresponding Fourier-transformed image.

To verify the shrinkage of the micelles, we performed a secondary hydrothermal crystallization treatment and lowered the hydrothermal temperature. The sample, labeled MCM−L, which was hydrothermally synthesized at 150 °C for 24 h, was divided equally into two fractions: one fraction was subjected to direct hydrothermal crystallization at 102 °C for 24 h, labeled MCM−M, and the other fraction was added 4 mL of water glass and subjected to secondary hydrothermal crystallization at 102 °C for 24 h, labeled MCM−N. Sample designations and hydrothermal treatment conditions are given in Table 2. Figure 4 illustrates TEM images and their corresponding Fourier-transformed images of samples post hydrothermal treatment at 150 °C (a) without additional water glass (b) and with 4 mL of water glass added, followed by further treatment at 100 °C for 24 h (c). All samples exhibit clear hexagonal pore channels, with the pore arrangement following the p6 mm pattern, indicating a well-maintained material structure. The silica source enters the pore channels through the hydration layer between the micelle and silica interface and condenses on the pore walls.

**Figure 5:**
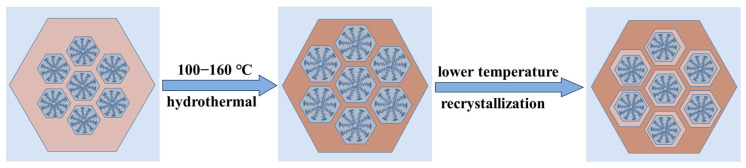
The effect of the thermal expansion of micelles at lower temperatures on MCM−41.

The SAXD results (Figure S3b) reveal no significant changes in the (100) plane spacings of the materials. This is attributed to micelle contraction, which does not alter the precipitation–dissolution equilibrium of silica at the interface. As a result, the lattice movement is halted. The addition of 4 mL of water glass introduces a substantial number of oligomeric silicates, which condense in the gaps between the pore wall interface and micelles. Due to the mismatch in size between the pore walls and the contracting micelles inside, the condensed precipitated silica cannot be uniformly distributed on the surface of the pore walls. This results in reduced orderliness of the pore channel arrangement after secondary crystallization, as evidenced in the SAXD spectra by a decrease in diffraction peak intensity and an increase in half-peak width. The surface area and mesopore analysis results of the three samples, as shown, indicate a slight increase in pore size after secondary hydrothermal treatment at 100 °C. This is due to the reduced protective effect of the micelles on the mesoporous channel interface after contraction, akin to the changes observed in MCM−41 materials post-surfactant removal and hydrothermal treatment [28]. The silica at the pore wall interface dissolves. The material with 4 mL of added water glass shows a significant reduction in pore size to 3.20 nm, almost equal to the pore size obtained by direct hydrothermal synthesis at 100 °C. These results conclusively demonstrate that the enlargement of MCM−41 material pore size with increasing hydrothermal temperature is due to the thermal expansion of micelles from low to high temperatures, and this expansion is reversible, with micelles contracting from high to low temperatures. The effect of the above thermal expansion of micelles at lower temperatures on MCM−41 can be illustrated in schematic Figure 5.

**Figure 6:**
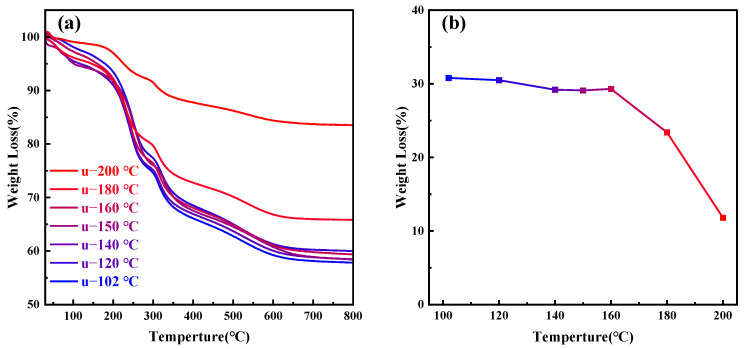
(**a**) Thermogravimetric (TG) analysis graphs of samples treated at different hydrothermal crystallization temperatures; (**b**) CTAB content in samples at different hydrothermal crystallization temperatures.

As the temperature continues to rise above 180 °C, the orderliness of MCM−41 materials significantly decreases; at 200 °C, it completely loses its orderliness, indicating that CTAB molecules cannot aggregate to form a two-dimensional hexagonal liquid crystal phase at 200 °C. Figure 6a presents the thermogravimetric analysis of MCM−41 samples synthesized but uncalcined at different hydrothermal crystallization temperatures, while Figure 6b depicts the weight loss percentage of the materials between 200–550 °C, indicative of the CTAB content. The primary weight loss components from room temperature to 200 °C are adsorbed and bound water in the material, while post 550 °C, the main weight loss is due to water produced from the condensation of silanol groups [26]. It is evident that post 180 °C, the CTAB content in the material significantly decreases. Other researchers have also observed comparable experimental results, with the proportion of silica in synthesized MCM−41 materials gradually increasing with temperature [28,44]. Since the total amount of silica source added to the solution remains constant, the quantity of silica in MCM−41 materials is fixed, with only the degree of silica condensation varying. Therefore, the reduction in CTAB content is due to its dissolution from the mesoporous channels, failing to serve as an ordered template for silica, ultimately leading to the loss of material orderliness.

Shah et al. determined the maximum surface excess concentration (Γ_max_) variation with temperature by measuring changes in the surface tension of CTAB solutions, as depicted in Figure S4 [45]. It is observed that Γ_max_ gradually decreases with increasing temperature. The maximum surface excess concentration at the air/water interface, Γ_max_, is calculated using the Gibbs adsorption isotherm [46]:

(4) $$\Gamma_{\max}=-\frac{1}{2.303\mathrm{nRT}}{\;[\frac{d\gamma }{\mathrm{dlogC}}]}_{T,P}\;,$$

where n = 1 for non-ionic surfactants and n = 2 for monovalent ionic surfactants, R is the ideal gas constant (8.314 J·mol^−1^·K^−1^), T is the absolute temperature, C is the surfactant concentration, and dγ/dlogC is the slope of the surface tension γ of the surfactant solution versus logC after the CMC.

According to Israelachvili et al. [47], the packing parameter p of surfactants is defined as

(5) $$P=\frac{V_{0}}{A_{\min}l_{c}}$$

For spherical micelles, P < 1/3; for cylindrical micelles, 1/3 < P < 1/2; for cubic phases, 1/2 < P < 2/3; and for lamellar liquid crystals, P > 1. $A_{min}$ is the minimum area per surfactant molecule at the air/water interface, reached at the CMC, is calculated as

(6) $$A_{min}=\frac{1}{N\Gamma_{max}},$$

where N is Avogadro’s number. According to this formula, $A_{min}$ increases as Γ_max_ decreases, ultimately reducing the packing parameter P [48].

**Figure 7:**
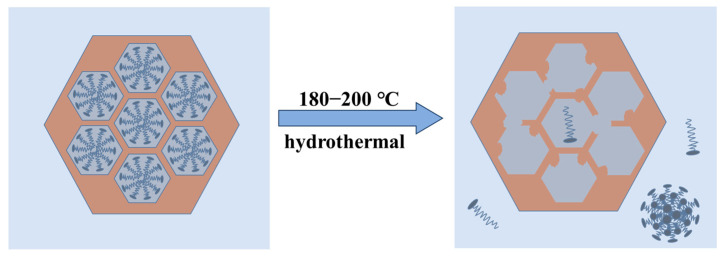
Loss of the mesoporous structure in MCM−41 material at higher hydrothermal crystallization temperatures.

The SAXD results (Figure 1) and TEM images of samples synthesized at 180 °C and 200 °C (Figure S5) indicate a gradual loss of 2D hexagonal arrangement (p6 mm) order in the samples. It can be inferred that in this system, when the temperature exceeds 180 °C, the packing parameter P of the micelles decreases to less than 1/3, favoring the formation of spherical micelles. However, these spherical micelles cannot act as ordered templates. According to Tornblom et al.’s measurements of CTAB’s second critical micelle concentration and Bergstrom’s classification of micelle aggregation states, the transition of micelles from hexagonal arrangement to disordered spherical micelles is a sudden process [49,50]. Therefore, the disappearance of the mesoporous structure occurs within a narrow temperature range, and no significant decrease in orderliness was observed under 100–160 °C conditions. When CTAB molecules detach from the channels, the H_2_O at the MCM−41 interface at high temperatures attack the Si−O−Si bonds, dissolving them into oligomeric silicates, which eventually condense into silica particles in water or re-precipitate on the existing silica surface. The change of MCM−41 material at higher hydrothermal crystallization temperature can be shown in Figure 7.

## 4. Conclusions

This investigation delves into how temperature variations during the hydrothermal synthesis of MCM−41 mesoporous silica influence pore size and structural order. As the temperature rises from 100 °C to 160 °C, the micelles’ stacking parameter P remains between 1/3 and 1/2, sustaining a stable hexagonal arrangement with thermal expansion causing an increase in diameter. Consequently, the pore size of MCM−41 expands from 3.17 nm to 3.45 nm, and the lattice constant grows from 4.50 nm to 4.96 nm, while maintaining a high degree of pore orderliness. This expansion is reversible; when the temperature is lowered and an appropriate amount of water glass is added, the micelles contract, reducing the pore size accordingly. This indicates that the pore diameter and wall structure of MCM−41 can be finely tuned by precise control of the synthesis conditions. However, at temperatures exceeding 180 °C, the micelles fail to maintain their ordered arrangement, and the accelerated dissolution of silica leads to structural collapse. At 200 °C, the MCM−41 material exhibits a significant decrease in structural order, completely losing its pore structure, with the specific surface area significantly reduced from 1011 m^2^/g at 100 °C to 307 m^2^/g. These findings suggest that precise control within the appropriate hydrothermal temperature range can optimize the pore size and structure of MCM−41 materials, providing tailored material designs for applications in fields such as petroleum cracking catalysis and drug delivery. Furthermore, changing the silica source during secondary hydrothermal treatment may play a role in improving pore surface properties, offering new avenues for enhancing material functionalization.

## Figures and Tables

**Table 1:** Structural properties of samples synthesized and calcined at different temperatures (T).

T	$S_{BET}^{a}$ (m^2^·g^−1^)	$D_{P}^{b}$ (nm)	$V_{P}^{c}$ (cm^3^·g^−1^)	$D_{H}^{d}$ (nm)	$d_{100}^{e}$ (nm)	$a_{0}^{f}$ (nm)	$W^{g}$ (nm)
102 °C	1011	3.17	1.11	3.02	3.89	4.50	1.48
120 °C	980	3.23	1.01	3.08	3.93	4.54	1.52
140 °C	945	3.37	0.97	3.21	4.12	4.76	1.55
150 °C	936	3.43	0.92	3.27	4.15	4.79	1.52
160 °C	890	3.45	0.91	3.29	4.3	4.96	1.67
180 °C	657	3.86	0.82	3.68	5.05	5.83	2.15
200 °C	307	—			—	—	

*^a^* BET specific surface area; *^b^* Most probable pore diameter; *^c^* Total pore volume; *^d^* Hexagonal aperture equivalent to $D_{P}$; *^e^* (100) Crystal plane spacing; *^f^* Lattice constant; *^g^* Wall thickness.

**Table 2:** The labeled sample designations and their hydrothermal conditions (Each hydrothermal session lasts 24 h).

Sample Designation	Primary Hydrothermal	Secondary Hydrothermal
MCM−J	120 °C	—
MCM−K	120 °C	180 °C
MCM−L	150 °C	—
MCM−M	150 °C	102 °C
MCM−N	150 °C	102 °C, 4 mL water glass

## Data Availability

The data were included in the text.

## Supplementary Materials

The following supporting information can be downloaded at: https://www.mdpi.com/article/10.3390/ma17081711/s1, Figure S1. FTIR spectra of (a) MCM−41 samples after hydrothermal crystallization at various temperatures; (b) CTAB under different treatment conditions; Figure S2. (a) Variation of the conductivity of CTAB solutions with concentration at different temperatures; (b) Changes in CMC of CTAB aqueous solutions with temperature; Figure S3. SAXD spectra. (a) MCM−J and MCM−K; (b) MCM−L, MCM−M and MCM−N; Figure S4. TEM image of the sample after different hydrothermal treatments. (a) 180 °C; (b) 200 °C; Figure S5. The variation of $\Gamma_{\max}$ with temperature.
