# Supplementary material for: Temperature-Driven Structural Evolution during Preparation of MCM−41 Mesoporous Silica
Source: Materials (Basel). 2024 Apr 9;17(8):1711. doi: 10.3390/ma17081711 (PMC11051505; doi:10.3390/ma17081711)
Supplement: Supplementary file 1 [file materials-17-01711-s001.zip › materials-2932677-supplementary.pdf]

Supplementary Materials

Temperature-Driven Structural Evolution during Preparation of MCM–41 Mesoporous Silica

Tao Xu [1], Kuixin Cui [1,2] and Shengming Jin [1,2,*]

[1] School of Minerals Processing and Bioengineering, Central South University, Changsha 410083, China;
xu-tao@mail.csu.edu.cn (T.X.); kuixin.cui@csu.edu.cn (K.C.)

[2] Key Laboratory for Mineral Materials and Application of Hunan Province, Central South University, Changsha 410083, China

* Correspondence: shmjin@csu.edu.cn

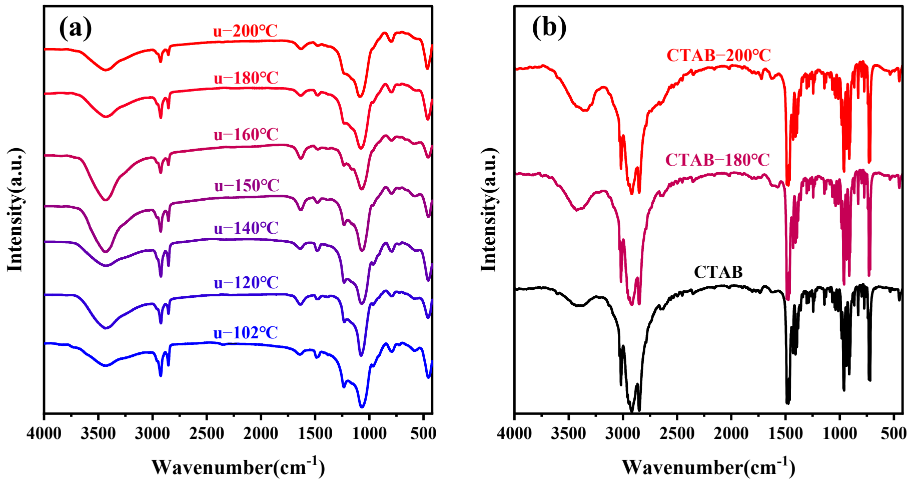

Figure S1. FTIR spectra of (a) MCM−41 samples after hydrothermal crystallization at various temperatures; (b) CTAB under different treatment conditions.

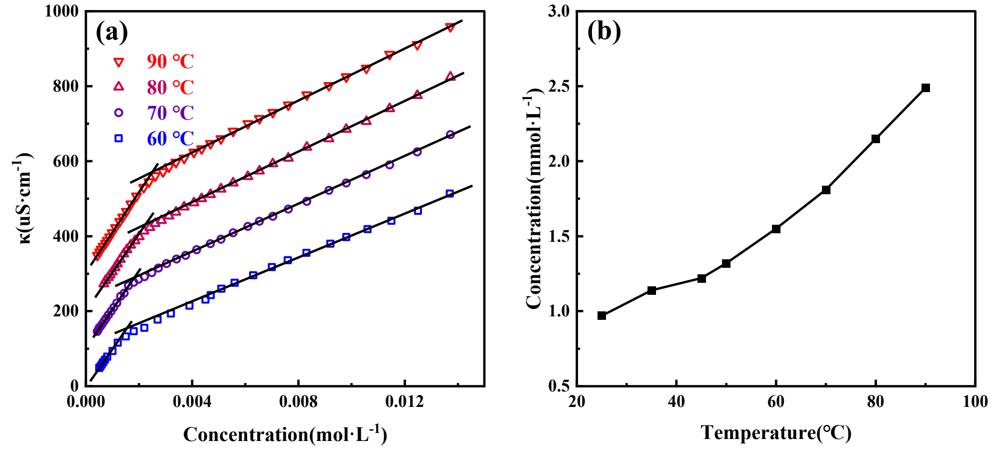

Figure S2. (a) Variation of the conductivity of CTAB solutions with concentration at different temperatures; (b) Changes in CMC of CTAB aqueous solutions with temperature.

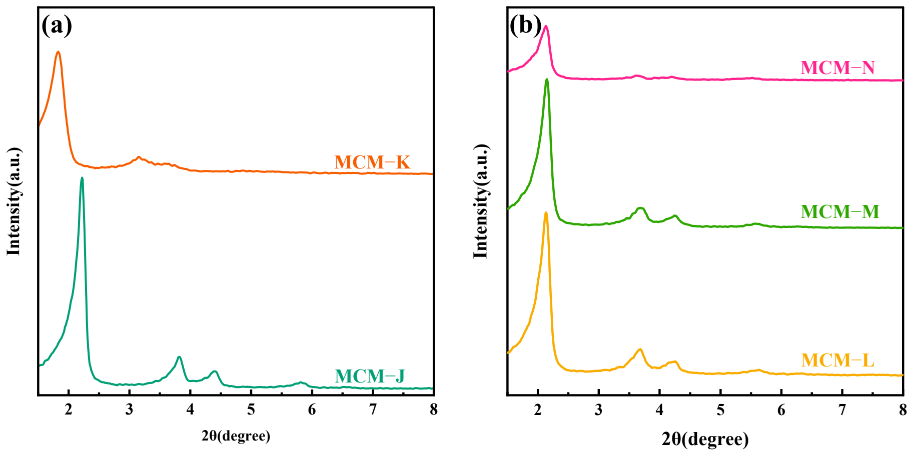

Figure S3. SAXD spectra. (a) MCM−J and MCM−K; (b) MCM−L, MCM−M and MCM−N.

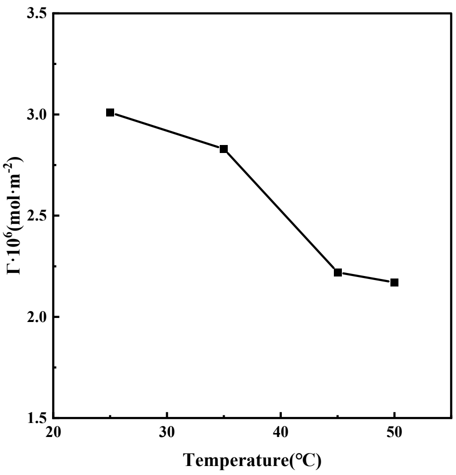

Figure S4. The variation of Γ_{max} with temperature.

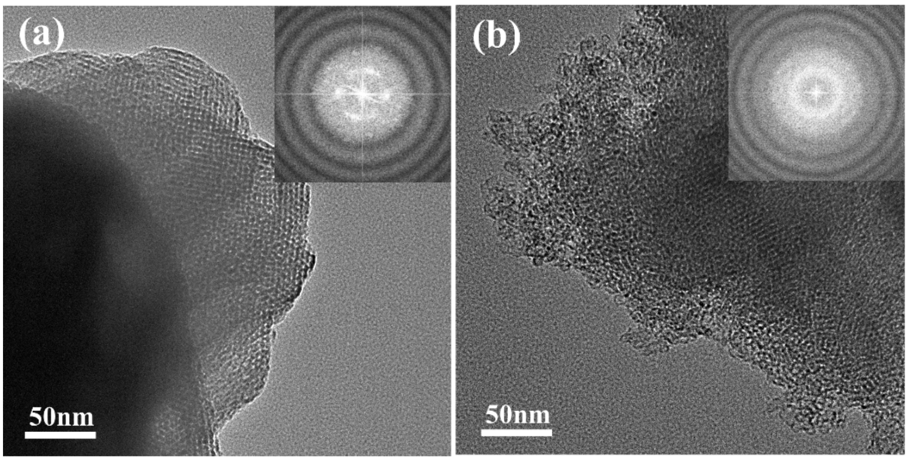

Figure S5. TEM image of the sample after different hydrothermal treatment. (a) 180 °C; (b) 200 °C.
